# The Utility of Colistin in Multiple Drug-Resistant *Pseudomonas aeruginosa* Bacterial Keratitis in a Kaposi’s Sarcoma Patient

**DOI:** 10.4274/tjo.galenos.2019.79999

**Published:** 2019-09-03

**Authors:** Özlem Barut Selver, Sait Eğrilmez, Samir Hasanov, Medine Yılmaz Dağ, Alper Tunger

**Affiliations:** 1 Ege University Faculty of Medicine, Department of Ophthalmology, İzmir, Turkey; 2 Ege University Faculty of Medicine, Department of Microbiology, İzmir, Turkey

**Keywords:** Colistin, multiple drug-resistant Pseudomonas aeruginosa, keratitis

## Abstract

A 71-year-old male patient presented with decreased visual acuity, redness, and discharge in his right eye for 5 days. He had undergone evisceration of his left eye several years earlier. Before presentation, he had received chemotherapeutic agents for Kaposi’s sarcoma of the scalp. Slit-lamp examination revealed severe hypopyon and an extensive corneal ulcer with surrounding infiltrate, which extended to the deep stroma. Microbiological evaluation identified the causative agent to be multiple drug-resistant *Pseudomonas aeruginosa*. Based on culture and susceptibility results, the patient was started on topical colistin 0.19% instilled hourly. Complete resolution of keratitis with residual corneal scarring was observed. In recent years, there has been an increase in drug resistance in *P. aeruginosa* keratitis. The lack of new antimicrobial agents against these resistant strains has led clinicians to reconsider colistin, which is an old drug. In this report, we aimed to stress the utility of colistin in multiple drug-resistant *P. aeruginosa* bacterial keratitis in a Kaposi’s sarcoma patient.

## Introduction

Microbial keratitis is one of the most important causes of corneal blindness.^1^ *Pseudomonas* *aeruginosa* as a bacterial etiological agent for microbial keratitis can cause severe clinical presentation.^2^ Drug resistance in ocular infections caused by *P. aeruginosa* was not common previously, but an increase in drug resistance in *P. aeruginosa* keratitis has been reported in recent years.^3,4,5,6^

Colistin (polymyxin E) is an old polypeptide antibiotic that mainly acts on the bacterial cell membrane and has outstanding in vitro activity against gram-negative bacilli. It currently has very limited systemic usage because of its potential nephrotoxicity and neurotoxicity.^7^ Topical usage that avoids the systemic side effects of colistin was reported in only a few articles.^8,9,10^

In this case report, we describe the therapeutic outcome of colistin, which is an old drug, for multiple drug-resistant (MDR) *P. aeruginosa* bacterial keratitis in a monocular Kaposi’s sarcoma patient. Our aim was to emphasize the risk factors for drug resistance for *Pseudomonas* keratitis, such as compromised immune system, and the importance of using targeted medication to control the disease.

## Case Report

**Figure 1:**
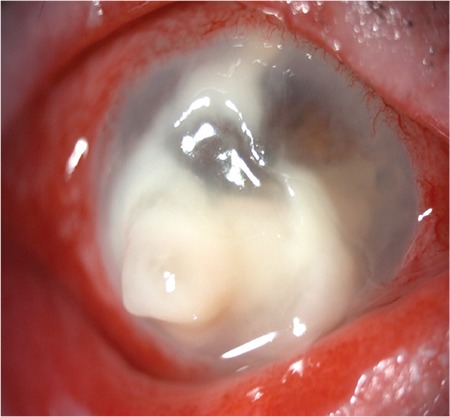
An extended corneal ulcer with surrounding infiltrate, which was extended to the deep stroma with severe hypopyon

A 71-year-old man presented with decreased visual acuity, redness, and discharge in his right eye for the last 5 days. He had undergone evisceration of his left eye (unknown etiology) several years earlier. Before presentation, he had received chemotherapeutic agents for Kaposi’s sarcoma of the scalp. Visual acuity in his right eye was light perception. Slit-lamp examination revealed severe hypopyon and an extensive corneal ulcer with surrounding infiltrate which extended to the deep stroma (Figure 1).

**Figure 2:**
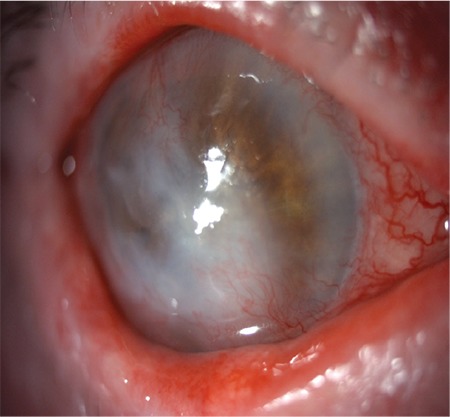
Complete resolution of keratitis with residual corneal scarring

Fundus visualization was not possible, but B-scan ultrasound revealed a normal posterior segment. After epithelial scraping was taken and sent to the laboratory for culture, empirical antibiotherapy (fortified topical antibiotics: vancomycin 50 mg/mL, ceftazidime 50 mg/mL hourly) was started. Microbiological evaluation identified the causative agent to be MDR *P. aeruginosa.* Based on culture and susceptibility reports (resistant to tobramycin, netilmicin, piperacillin/tazobactam, cefepime, imipenem, meropenem, amikacin, gentamicin, ciprofloxacin, and ceftazidime; sensitive to colistin), previous empirical treatment was stopped and the patient was started on hourly instillation of topical colistin 0.19% with no systemic antibiotic until clinical regression was achieved. Three days after initiating hourly topical colistin, dosing was tapered first to every 2 hours, then to every 3 hours at 1 week, and to every 6 hours after 10 days. Topical colistin was continued every 6 hours for 1 month after the first diagnosis. Complete resolution of keratitis with residual scarring was noticed at 3 weeks (Figure 2). Renal function was assessed with blood urea nitrogen and serum creatine before topical colistin and weekly after treatment to monitor for nephrotoxicity. For ocular tolerance and toxicity, the gradual decreases in symptoms such as burning and stinging and signs such as conjunctival hyperemia, which existed before topical colistin treatment, were accepted as safety indicators and were examined repeatedly during treatment, first daily and later weekly.

During the hospitalization period, the oncology and plastic surgery departments were consulted and no additional chemotherapeutic or immunomodulatory agents were applied in accordance with these consultations.

**Figure 3:**
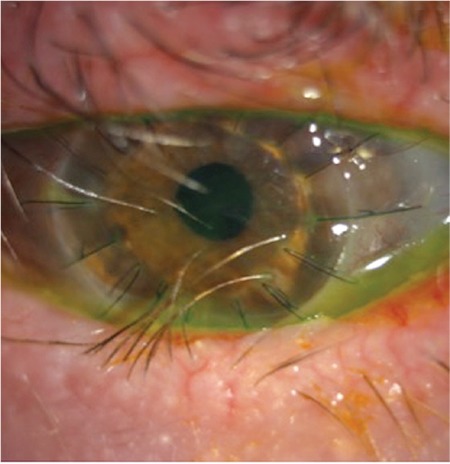
Clear corneal graft

Penetrating keratoplasty was performed 5 months after presentation. In follow-up examination on postoperative day 3, resolution of the corneal edema was observed (Figure 3) and best-corrected visual acuity (BCVA) was 20/400. BCVA remained stable during follow-up (6 months) with no recurrence of infection.

## Discussion

Antimicrobial classes with activity against *P. aeruginosa *are the aminoglycosides, anti-pseudomonal/carbepenems/cephalosporins/fluoroquinolones/penicillins + b lactamase inhibitors, monobactams, phosphonic acids, and polymyxins. MDR *P. aeruginosa* is defined as lack of sensitivity to one or more agents in at least three antimicrobial categories.^11^

Recently, there has been an increase in drug resistance in *P. aeruginosa *keratitis.^12^ Drug-resistant *P. aeruginosa *keratitis is a therapeutic challenge because of the lack of medications. This has led clinicians to the reconsideration of colistin, which is an old drug.^13^

Colistin was discovered in 1949^14^ and its parenteral form was used extensively in the 1960s. The drug was then gradually abandoned in the early 1980s because it induced nephrotoxicity.^15,16,17,18^ Given its excellent activity against a variety of gram-negative bacilli, colistin was later reconsidered for the treatment of systemic infections due to the rising number of infections caused by MDR gram-negative bacteria in recent years.^19,20^

According to our literature search, there are only a few published articles reporting the topical use of colistin.^8,9,10^

In our case, antibiogram results indicated that the isolate was resistant to all antibiotics except colistin. Therefore, we opted to administer colistin as a topical treatment. Based on the antibiotic susceptibility tests, this *P. aeruginosa* strain was considered MDR because of the lack of sensitivity to at least one agent in at least three antimicrobial categories, as described above. A drug concentration of 0.19% was chosen for topical usage, in accordance with the literature.^8,21^

Moreover, most patients had either ocular (contact lens use, topical corticosteroids, ocular surface disorder) and/or systemic risk factors (leukemia, Stevens Johnson syndrome, diabetes mellitus) predisposing to microbial keratitis.^22,23,24^ In the present case, the patient’s history of Kaposi’s sarcoma was considered a major risk factor, as a systemic immunocompromising condition that facilitates microbial keratitis. To the best of our knowledge, this is the first case report of *P. aeruginosa* keratitis in a patient with Kaposi’s sarcoma who was treated with topical colistin.

In summary, we conclude from this case that the usage of topical 0.19% colistin for the treatment of MDR *P. aeruginosa* keratitis was an effective alternative that did not cause nephrotoxicity or ocular side effects. Because topical drug administration is the first and best method of keratitis treatment, the old and forgotten but still significantly effective agent colistin is a safe alternative that can be considered for MDR gram-negative bacterial keratitis. Further studies with larger sample sizes and control groups are needed to validate the efficacy of topical colistin.
